# Prospective observational study of the use of omeprazole and maropitant citrate in veterinary specialist care

**DOI:** 10.1038/s41598-020-72950-3

**Published:** 2020-09-25

**Authors:** Rachel McCormack, Louise Olley, Barbara Glanemann, James W. Swann

**Affiliations:** 1 Department of Clinical Science and Services, Royal Veterinary College, Hatfield, UK; 2 Kennedy Institute of Rheumatology, University of Oxford, Roosevelt Drive, Oxford, OX3 7FY UK

**Keywords:** Gastroenterology, Health care

## Abstract

The proton pump inhibitor omeprazole is administered to dogs with gastroduodenal ulceration or oesophagitis, whereas the neurokinin-1 receptor antagonist maropitant citrate is licensed as an antiemetic drug. In people, omeprazole is overprescribed in hospitals, increasing the risk of adverse effects and imposing unnecessary costs in healthcare. To investigate the use of omeprazole and maropitant in our veterinary specialist hospital, we conducted a prospective observational study in its Medicine and Surgery wards, recording patient data and obtaining contemporaneous information from clinicians about their reasons for administering either drug. In doing so, we find omeprazole and maropitant are administered to a large proportion of dogs, including to many of those with no presenting signs suggestive of gastrointestinal disease. We find prescribing clinicians consider both drugs safe but often underestimate their financial cost. We find the stated reasons and objective predictors of administration of both drugs vary according to clinical setting but that these modalities yield concordant results. Reviewing the manner of administration and stated indications for use of both drugs, we find omeprazole is often administered outside dosing recommendations, and both drugs are frequently administered for aims that are unlikely to be achieved when considering their known biological effects in dogs. In conclusion, our work reveals probable overprescribing of omeprazole and maropitant citrate in hospitalised dogs, highlighting a need for initiatives to decrease inappropriate prescribing.

## Introduction

Gastroprotectant (GP) drugs, including the proton pump inhibitor (PPI) omeprazole, increase the pH of gastric fluid, facilitating repair of damaged tissue in the stomach and proximal small intestine. Consequently, omeprazole is indicated in dogs for treatment of gastroduodenal ulceration, which may be caused by locally invasive neoplasia, hepatic disease, inflammatory bowel disease, ingestion of non-steroidal anti-inflammatory drugs (NSAIDs), or hyperstimulation of gastric acid production by gastrinomas^1–3^. Omeprazole is also indicated for treatment of oesophagitis, which is frequently caused or exacerbated by reflux of acidic gastric fluid into the oesophagus^4^. Similarly, pre-operative administration of omeprazole decreases the frequency of oesophageal low pH events caused by gastro-oesophageal reflux (GOR)^5^; this is probably because PPIs increase the pH of gastric fluid rather than because they decrease the frequency of reflux events^6^. However, it remains unclear whether prophylactic administration of omeprazole would prevent development of oesophagitis in dogs that have suffered transient vomiting or regurgitation. Routine use of omeprazole for treatment of acute, nonerosive gastritis in dogs is not recommended by a consensus panel, nor for prophylactic treatment of dogs with kidney disease that is not complicated by signs of gastrointestinal (GI) disease^4^. For a number of conditions, there is currently insufficient evidence to establish whether omeprazole produces a beneficial effect in dogs; these include GI haemorrhage related to thrombocytopenia and liver disease without GI signs.

The frequency of use of omeprazole in veterinary practice is unknown but, in human medicine, PPIs are among the most widely prescribed drugs, accounting for an estimated 113 million annual prescriptions in the USA^7^. In humans, PPIs are associated with possible adverse effects^8^, including bone fracture related to calcium malabsorption^9^, enteric bacterial infections^10^, and deficiencies of micronutrients such as vitamin B12^11^. Consequently, growing use of PPIs has been matched by concern about inappropriate prescriptions to patients who do not require them but could be exposed to their adverse effects^12^. Of particular concern is hospital administration of PPIs, with previous studies showing that 30–60% of such prescriptions are inappropriate and that patients frequently continue to take PPIs beyond their hospital stay owing to failure of deprescription^13–16^. In veterinary medicine, although the incidence of adverse effects attributable to GPs is unknown and probably lower than in people, guidelines have been advanced for their use based on clinical evidence and expert opinion^4^. However, in our experience, PPIs are often used outside these recommendations in dogs, leading us to ask whether the rate of inappropriate prescriptions could be similar to that reported in people. If so, this practice could expose dogs to the risk of adverse effects, drug interactions, prescribing errors, and accidental overdoses, and also impose an unnecessary financial cost on their owners^17^.

In addition to GPs, many dogs receive antiemetic treatment with the drug maropitant citrate, which acts as an antagonist for the neurokinin-1 receptor, inhibiting the actions of substance P and other tachykinins that may induce emesis. Maropitant, which is similar to aprepitant and fosaprepitant used in people, has been licensed for treatment of vomiting in dogs in the UK^18, 19^. Previous studies have confirmed it effectively reduces the occurrence of vomiting caused by chemotherapy^20, 21^, morphine^22^, and motion sickness in dogs^23^, but its effect on nausea has been more difficult to examine, partly owing to the notorious difficulty in evaluating this problem in animals^24^. In previous investigations, maropitant had no effect on the frequency of nausea-associated behaviours, such as ptyalism, lip-licking, and swallowing, in dogs receiving morphine^22^ or undergoing general anaesthesia^25^, and was less effective for this purpose than the 5HT_3_ receptor antagonist ondansetron^26^. Nevertheless, it has been our impression that maropitant is administered frequently in veterinary hospitals for treatment of vomiting and perceived nausea, leading us to ask, as with PPIs, whether this treatment was appropriate in all cases.

In this study, we sought to investigate (1) how frequently GPs and antiemetic drugs were administered to dogs presented with or without signs of GI disease, (2) what were the indications and risk factors for administration of omeprazole and maropitant in dogs, particularly when this was initiated in cases not presented for treatment of GI disease, (3) whether prescriptions of omeprazole and maropitant were appropriate when considered against published recommendations for their use, and (4) whether major patterns of and reasons for use differed between Medicine and Surgery wards of a veterinary hospital.

## Results

### Population characteristics

To investigate the use of GP and antiemetic drugs in veterinary specialist care, we conducted a prospective observational study in the Medicine and Surgery wards of our hospital. Data relating to administration of these drugs and observations on the clinical course of the patient were recorded for all dogs hospitalised for at least 24 h. In the periods of data collection, there were 261 eligible admissions to the Medicine ward and 146 to the Surgery ward. Among these, 24 admissions were excluded from further analysis because they represented repeated visits of dogs presented previously in the study period (22 in Medicine ward and 2 in Surgery ward). The reasons for presentation of dogs in both wards are shown in Supplementary Table S1.

**Figure 1:**
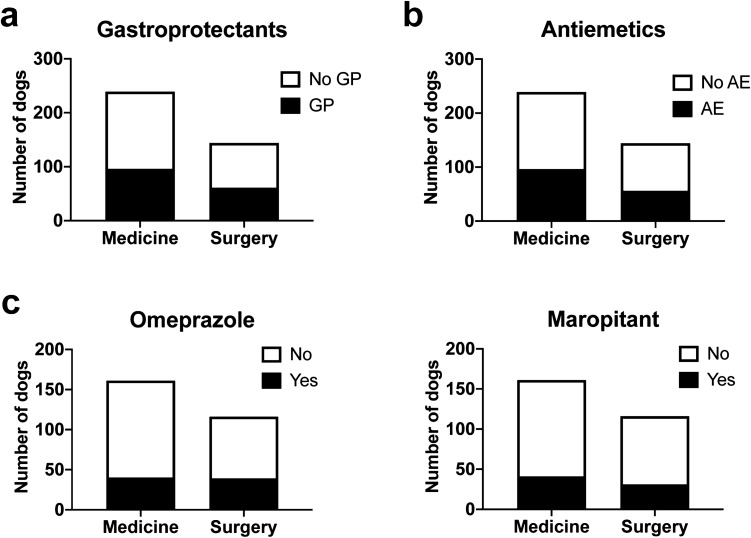
Gastroprotectants and antiemetics are administered frequently but with no difference between wards. Number of dogs receiving gastroprotectant (GP) drugs (**a**) or anti-emetics (AE) (**b**) during hospitalisation. Also shown are number of dogs presented without signs of gastrointestinal disease that received omeprazole or maropitant citrate (**c**).

Among admitted cases, 96 dogs in Medicine ward (n = 239, 40.2%, 95% confidence interval [CI]: 34.2–46.5) and 61 dogs in Surgery ward (n = 144, 42.4%, 95% CI: 34.6–50.5) received at least one dose of a GP drug (comprising omeprazole, sucralfate, ranitidine, or misoprostol), with no difference in proportion between wards (Chi squared 0.179, p = 0.672) (Fig. 1a). Similarly, there was no difference in the proportion of dogs in each ward receiving an antiemetic drug (comprising maropitant, ondansetron, or metoclopramide; Medicine: 96/239, 40.2%, 95% CI: 34.2–46.5; Surgery 56/144, 38.9%, 95% CI: 31.3–47.0; Chi squared 0.06, p = 0.804) (Fig. 1b). The frequency of administration of individual drugs in each ward is shown in Tables 1 and 2. Owing to the frequency of their use, we focused the remainder of our analysis on omeprazole and maropitant, though we present data relating to use of ondansetron in Supplementary Table S2.

**Table 1:** Frequency of use of gastroprotectant drugs in hospitalised dogs.

Drug	Ward	
	Medicine (n = 239) N (%)	Surgery (n = 144) N (%)
Omeprazole	96 (40.2)	61 (42.4)
Ranitidine	1 (0.4)	0
Sucralfate	8 (3.3)	3 (2.1)
Misoprostol	3 (1.3)	0

**Table 2:** Frequency of use of antiemetic drugs in hospitalised dogs.

Drug	Ward	
	Medicine (n = 239) N (%)	Surgery (n = 144) N (%)
Maropitant citrate	89 (37.2)	52 (36.1)
Ondansetron	41 (17.2)	1 (0.7)
Metoclopramide	9 (3.8)	12 (8.3)

### Omeprazole is administered frequently and considered safe and affordable by prescribing veterinarians

The most commonly prescribed GP drug in both wards was omeprazole, which was usually administered intravenously (133/156 dogs, 85.3%) and twice daily (145/156 dogs, 92.9%) at a median dosage of 2 mg/kg per day (inter-quartile range [IQR]: 2–2). In many cases in both wards, omeprazole was prescribed to dogs that were presented to the hospital with some combination of vomiting, regurgitation, or melaena, even if this was not the primary reason for referral. This occurrence was more common in Medicine than Surgery ward (Medicine 56/96, 58.3%; Surgery 22/61, 36.1%; Chi squared 7.398, p = 0.007) (Fig. 1c), which probably reflects the difference in caseload between these clinical disciplines.

Because we were surprised by the frequency of its use in hospitalised dogs, we enquired among clinicians about their general attitudes towards the use of omeprazole. Most considered it a safe or very safe drug (median score 2, IQR: 2–3, on a scale of -3 “very unsafe” to + 3 “very safe”, n = 14), but 7/14 (50%) believed incorrectly that it has a marketing authorisation for dogs in the UK. As a further possible factor that might affect its widespread use, we asked clinicians to estimate the cost of administering omeprazole for 1 day to a 10 kg dog. In their responses, most (9/14, 64.3%) underestimated the true cost of £15.64. Collectively, this showed omeprazole is prescribed widely by clinicians who have few concerns about its safety and who underestimate its cost.

### In hospitalised dogs presented without GI signs, omeprazole is predominantly administered in response to new signs

We wished to understand why omeprazole was being prescribed to hospitalised dogs that had not been presented with GI signs that might prompt its use. To investigate this, we asked clinicians to complete a questionnaire stating their indications for administration of omeprazole in each dog. Importantly, clinicians were prompted to complete the questionnaire in their own words within 1 week of prescribing the drug, meaning the information provided was contemporaneous to the clinical decision. The response rate to these questionnaires was good, with no difference between wards (Medicine 28 responses/40 dogs that received omeprazole without a prior history of vomiting, regurgitation, or melaena, 70.0%; Surgery 24/39, 61.5%, Chi squared 0.629, p = 0.428). We coded the responses from questionnaires into similar domains because some respondents provided multiple reasons for use of omeprazole; here, we report the frequency of these different domains.

Stated indications for use of omeprazole by clinicians are shown in Table 3. In both wards, occurrence of vomiting and regurgitation while dogs were hospitalised was the most frequent indication when combined. Among Medicine clinicians, presence of a concurrent disease was noted as a contributory factor, consisting of azotaemia (n = 3), liver disease (1), pancreatitis (1), and inflammatory bowel disease (1). Additionally, a small proportion (14.3%) of respondents from the Medicine ward reported decreased appetite as a reason for administering omeprazole. In the Surgery ward, prophylactic treatment aiming to prevent GOR under general anaesthesia was frequently recorded as a reason for administering omeprazole, particularly for brachycephalic dogs undergoing surgery of the upper respiratory tract (37.5%).

**Table 3:** Stated indications for administration of omeprazole in dogs with no history of GI signs on presentation.

Indication	Ward	
	Medicine (N = 28) N (%)	Surgery (N = 24) N (%)
Vomiting while hospitalised	7 (25.0)	3 (12.5)
Regurgitation while hospitalised	4 (14.3)	9 (37.5)
Diarrhoea while hospitalised	0	2 (8.3)
Decreased appetite while hospitalised	4 (14.3)	0
Signs of nausea while hospitalised	2 (7.1)	3 (12.5)
Signs of GI haemorrhage or ulceration	4 (14.3)	0
Concurrent disease	6 (21.4)	0
Concurrent medications	3 (10.7)	2 (8.3)
Prophylactic treatment to decrease risk of gastro-oesophageal reflux under GA	0	10 (41.7), of which 9 (37.5) in dogs with BOAS

GA: general anaesthesia; BOAS: brachycephalic airway obstructive syndrome. Note that numbers summate to more than the number of questionnaire responses because some questionnaires contained multiple domains.

Because questionnaire responses could be subject to bias owing to observer effects and inaccurate recollection, we wished to triangulate our results with objective data collected by nursing staff from the same dogs while they were hospitalised. To do so, we constructed logistic regression models evaluating the association between ward observations, such as occurrence of GI signs, changes in appetite, and behavioural signs, with the decision to administer omeprazole during the hospital stay (Supplementary Table S3–S5). We produced separate models for Medicine and Surgery wards owing to the differences in the frequency of some potential risk factors, such as general anaesthesia.

**Figure 2:**
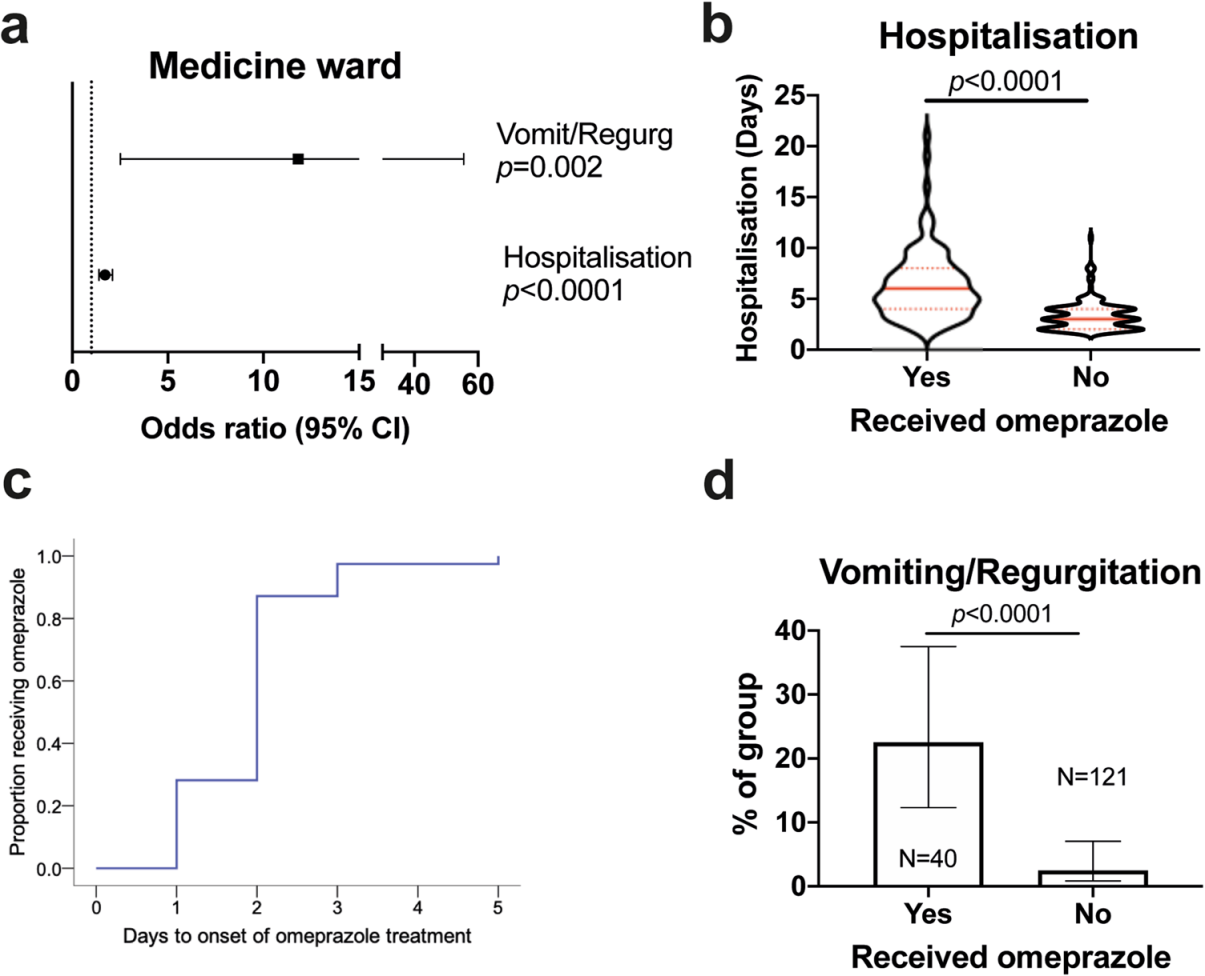
Development of vomiting or regurgitation is the major predictor of omeprazole administration in Medicine ward. (**a**) Forest plot showing odds ratios and 95% confidence intervals (CI) for variables significantly associated with omeprazole administration in multivariable binary logistic regression analysis. Vomit/regurg: vomiting or regurgitation. Dashed line indicates threshold. (**b**) Violin plot showing duration of hospitalisation in dogs that did or did not receive omeprazole. Solid red lines indicate median; dashed red lines indicates 1st and 3rd quartiles. Groups compared by Mann–Whitney U test. (**c**) Kaplan–Meier plot showing duration of time to onset of omeprazole administration in those dogs that received it (n = 39). (**d**) Proportion of dogs that had vomiting or regurgitation among those that did or did not receive omeprazole. Bars show proportion with 95% confidence intervals; groups compared by Chi squared test.

In the medicine ward, length of hospitalisation and occurrence of vomiting or regurgitation (before omeprazole was administered) were independently associated with administration of omeprazole (Fig. 2a); the interaction term for these variables was not significant. Examining these associations in more detail, we found that although vomiting/regurgitation in hospitalised dogs was not common, occurring in 12/161 dogs not admitted for GI disease, most of those affected went on to receive omeprazole (9/12, 75.0%) (Fig. 2b). Although length of hospitalisation was associated with omeprazole administration, the majority of dogs began to receive it within 1–2 days of admission (Fig. 2c). This suggests administration of omeprazole identifies those dogs destined to have a longer period of hospitalisation (Fig. 2d), rather than increasing as a function of hospitalisation.

**Figure 3:**
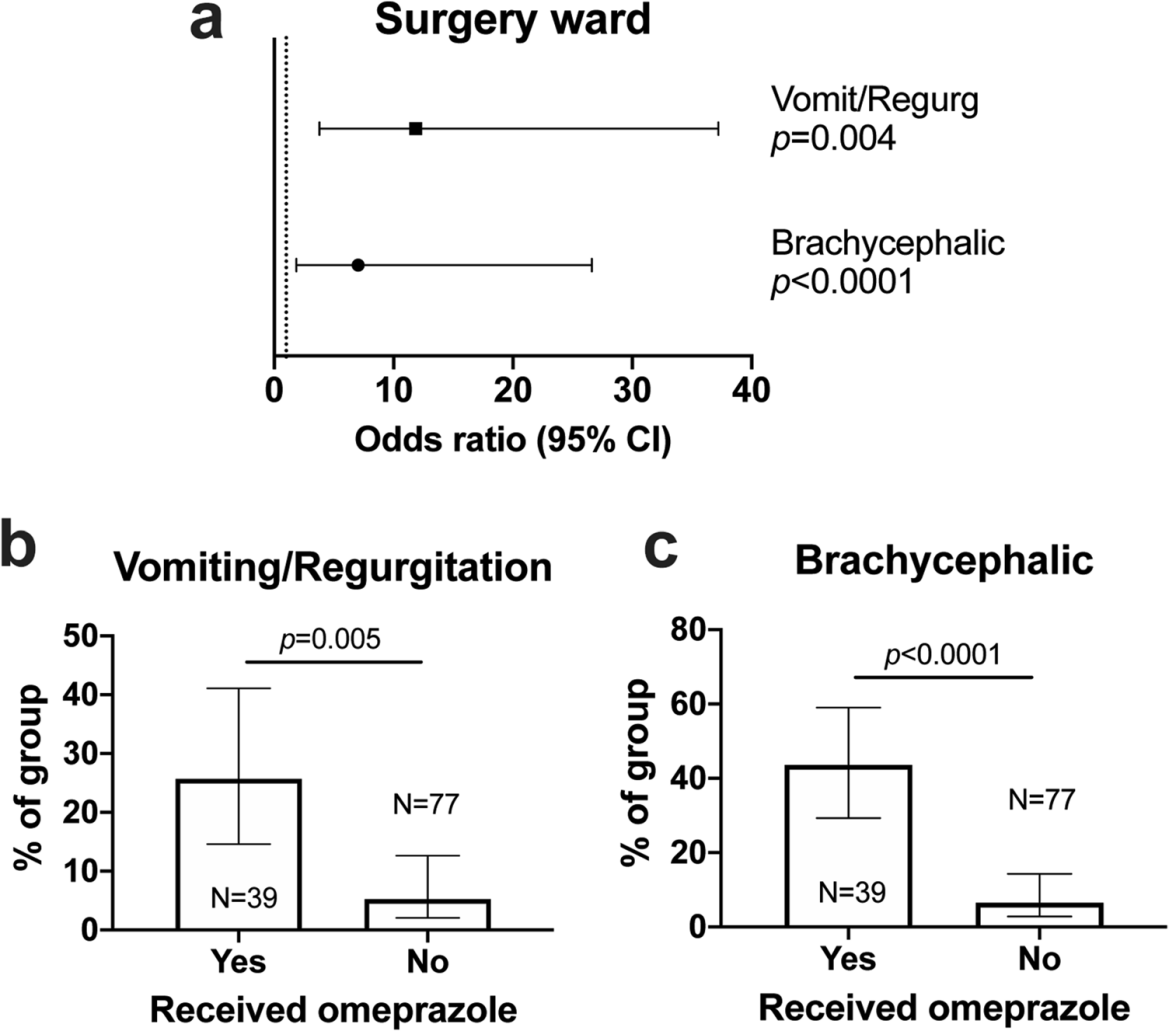
Membership of a brachycephalic breed and development of vomiting or regurgitation predict administration of omeprazole in Surgery ward. (**a**) Forest plot showing odds ratios and 95% confidence intervals (CI) for variables significantly associated with omeprazole administration in multivariable binary logistic regression analysis. Vomit/regurg: vomiting or regurgitation. Dashed line indicates threshold. (**b**) Proportion of dogs that had vomiting or regurgitation among those that did or did not receive omeprazole. Bars show proportion with 95% confidence intervals; groups compared by Chi squared test. (**c**) Proportion of dogs belonging to a brachycephalic breed among those that did or did not receive omeprazole. Bars show proportion with 95% confidence intervals; groups compared by Chi squared test.

Conducting the same analysis for dogs in the Surgery ward, we also found that vomiting or regurgitation increased the odds of omeprazole administration (Fig. 3a), with 10/14 (71.4%) dogs showing these signs going on to receive omeprazole after the episode (Fig. 3b). Additionally, we found dogs belonging to brachycephalic breeds were significantly more likely to receive omeprazole, regardless of their reason for presentation (Fig. 3c). Specifically, 17/22 (77.3%) brachycephalic dogs received omeprazole in the Surgery ward, compared to 5/11 (45.5%) of those in Medicine ward. Collectively, these models revealed striking concordance between indications stated by clinicians at the time of their clinical decision and objective data gathered in the wards: both modalities indicated the chief reason for administration of omeprazole in dogs presented without GI signs was for treatment of acute, hospital-acquired vomiting and/or regurgitation.

### Omeprazole is frequently administered outside dosing recommendations and for reasons incompatible with its known effects

Using recently published guidelines for the use of GP drugs in small animal practice that were based on the consensus recommendations of expert clinicians^4, 27^, we wished to determine whether prescriptions for omeprazole were appropriate. Accordingly, we assessed each case according to (1) drug factors, including the dosage and frequency of administration that should be achieved, and (2) patient factors, comprising indications stated by the clinician that were considered suitable for use of omeprazole (Table 4). Using these criteria, we found omeprazole was more commonly administered outside dosing recommendations in the Surgery ward than the Medicine ward (Surgery 13/59, 22.0%; Medicine 4/95, 4.2%; Fisher’s exact test, p = 0.005), which was almost equally attributable to administration of doses outside the recommended range by > 10% or to once daily administration for two or more days (Table 5). However, when evaluating patient factors, there was no difference between wards in the proportion of cases where omeprazole was administered for a reason unrelated to its expected effects (Medicine 27/63, 42.9%; Surgery 12/38, 31.6%, Chi squared 6.95, p = 0.139). Taken together, this indicated the manner of or stated reasons for administering omeprazole did not meet recommendations in a considerable proportion of cases in both wards, but the reasons for this differed between them.

**Table 4:** Drug and patient factors evaluated for inappropriate use of omeprazole and maropitant in dogs.

*Drug factors*			Considered inappropriate
Omeprazole	Route	IV or PO	No
	Frequency	BID	No
	Dosage	1 mg/kg per dose, with margin of 10% above or below	No
	Drug interactions	Concurrent administration with azole antifungal	Yes
Maropitant	Route	IV or PO	No
	Frequency	SID	No
	Dosage	1 mg/kg per dose if IV or 2 mg/kg if PO, with margin of 10% above or below	No
*Patient factors*			
Omeprazole	Indication	Vomiting if: Less than 3-week duration No evidence of haematemesis, melaena, or haemorrhage on endoscopy No concurrent regurgitation No evidence of ulceration on imaging	Yes
		Chronic vomiting, or vomiting with regurgitation	No
		Regurgitation with risk of oesophagitis	No
		Gastro-oesophageal reflux under GA	No
		Prophylactic administration before GA if reason to prevent gastro-oesophageal reflux	No
		Evidence of gastroduodenal ulceration or erosion (e.g. ulcer observed by endoscopy or imaging, haematemesis, melaena)	No
		Symptomatic hepatic disease*	No
		IRIS stage 1–3 chronic kidney disease without GI signs	Yes
		IRIS stage 1–3 chronic kidney disease with vomiting, regurgitation, or GI bleeding*	No
		IRIS stage 4 chronic kidney disease, with or without GI signs*	No
		Pancreatitis without evidence of gastroduodenal ulceration	Yes
		GI bleeding with thrombocytopenia*	No
		Anorexia or decreased appetite	Yes
		Administration of glucocorticoids at immunosuppressive dosage without GI signs or evidence of ulceration	Yes
		Administration of non-steroidal anti-inflammatory drugs in the absence of GI signs	Yes
		Diarrhoea without melaena or occult blood	Yes
		Indication not known	Yes
		Stated indication ‘continued from referring vet’	Yes
		Signs of nausea	Yes
Maropitant	Indications	Vomiting	No
		Signs of nausea	No
		Diarrhoea	Yes
		Gastro-oesophageal reflux under GA	Yes
		Prophylactic administration before GA if reason to anticipate gastro-oesophageal reflux	Yes
		Anorexia/decreased appetite	No
		Indication not known	Yes
		Stated indication ‘continued from referring vet’	Yes
		Regurgitation in the absence of vomiting	Yes
		Pancreatitis in the absence of vomiting, nausea, or decreased appetite	Yes

*Not considered inappropriate owing to insufficient published evidence on its use in this setting.

**Table 5:** Frequency of drug and patient factors for inappropriate use of omeprazole.

Factor	Ward	
	Medicine N with inappropriate use (%)	Surgery N with inappropriate use (%)
	N = 95	N = 59
*Drug factors*	4 (4.2)	13 (22.0)
Dose	2 (2.1)	6 (10.2)
Frequency	2 (2.1)	7 (11.9)
Route	0	0

	N = 63	N = 38
*Patient factors*	27 (42.9)	12 (31.6)
Diarrhoea	1 (1.6)	2 (5.3)
Decreased appetite	9 (14.3)	1 (2.6)
Continued from referring veterinarian	2 (3.2)	0
Vomiting unlikely to be responsive to omeprazole	10 (15.9)	2 (5.3)
Nausea	2 (3.2)	5 (13.2)
Immunosuppressive treatment	3 (4.8)	0
NSAID treatment	0	2 (5.3)

NSAID: non-steroidal anti-inflammatory drug.

As a final means to determine why clinicians in the Medicine ward might be administering omeprazole for the stated reason of decreased appetite, we provided two different case scenarios of dogs with this problem. The first described a dog with decreased appetite while hospitalised for lower urinary tract (i.e. non-GI) disease, while the second described a dog with pancreatitis that did not have vomiting/regurgitation upon presentation but did have abdominal pain and decreased appetite (i.e. GI-related). Administration of omeprazole to either dog would be ‘inappropriate’ according to our patient factors. In response to these scenarios, no Medicine clinicians opted to administer omeprazole to the non-GI dog, but 2/9 (22.2%) chose to administer it to the GI-related case, both citing a possible risk of vomiting and oesophagitis but not indicating they expected it to increase appetite. This suggested some willingness to implement treatment with omeprazole on a prophylactic basis, even in the absence of signs that it might be expected to affect.

### Maropitant is administered frequently in hospitalised dogs

In both wards, maropitant was usually administered intravenously (134/141, 95.0%) and at a dosage of 1 mg/kg once daily. When given orally (7/141 dogs), the median dosage was 1.9 mg/kg per day (IQR: 1.6–2.0). When asked about its administration, most respondents considered maropitant to be a safe or very safe drug (median score 2, IQR: 2–3, on a scale of − 3 to + 3, n = 14) and all correctly identified that it had marketing authorisation in the UK. However, most respondents (11/14, 78.6%) also underestimated the cost of administering the drug for one day in a 10 kg dog, which was £18.32.

**Figure 4:**
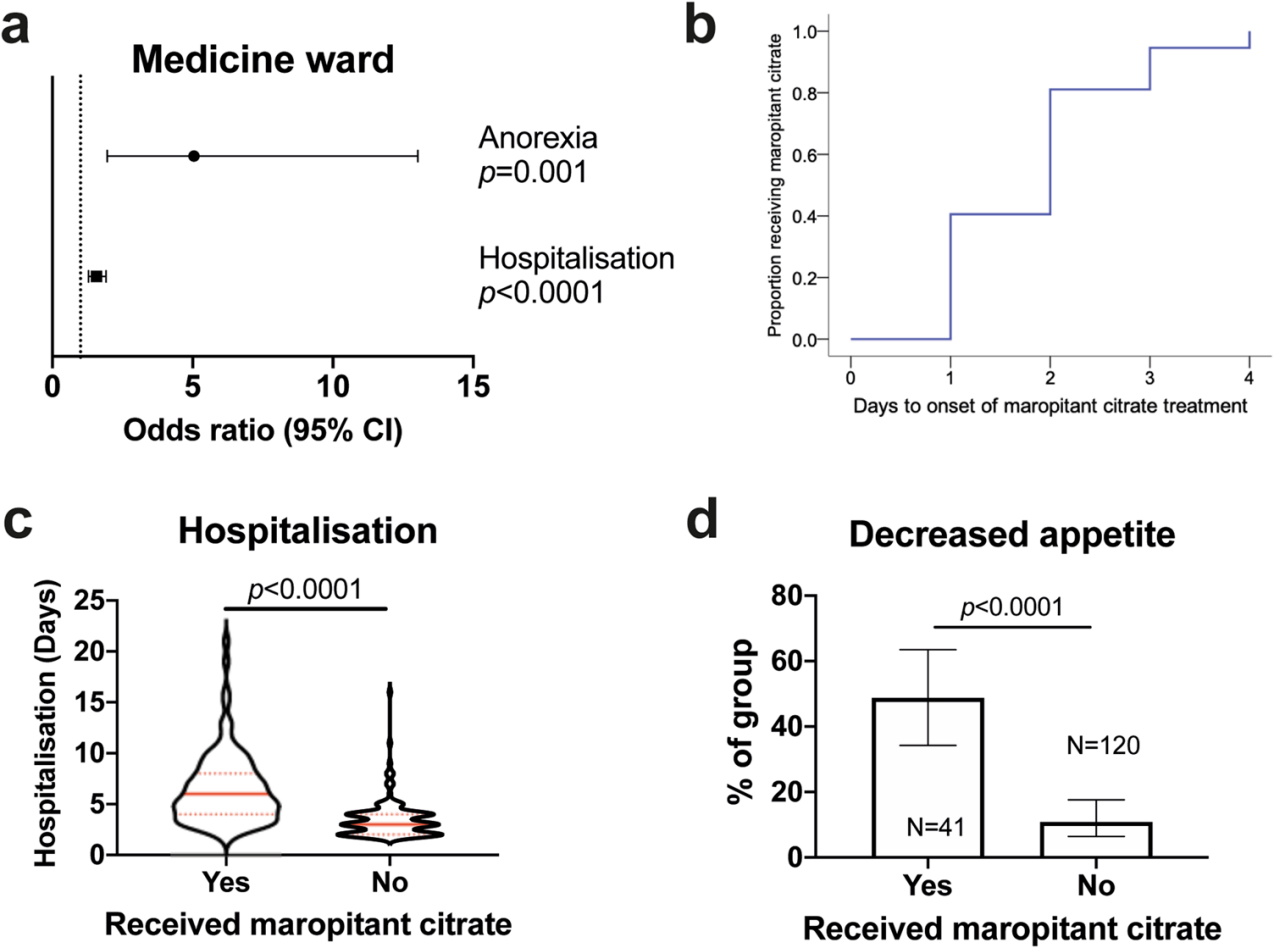
Duration of hospitalisation and decreased appetite predict administration of maropitant in Medicine ward. (**a**) Forest plot showing odds ratios and 95% confidence intervals (CI) for variables significantly associated with maropitant administration in multivariable binary logistic regression analysis. Dashed line indicates threshold. (**b**) Kaplan–Meier plot showing duration of time to onset of maropitant administration in those dogs that received it (n = 37). (**c**) Violin plot showing duration of hospitalisation in dogs that did or did not receive maropitant. Solid red lines indicate median; dashed red lines indicates 1st and 3rd quartiles. Groups compared by Mann–Whitney U test. (**d**) Proportion of dogs that had decreased appetite among those that did or did not receive maropitant. Bars show proportion with 95% confidence intervals; groups compared by Chi squared test.

**Figure 5:**
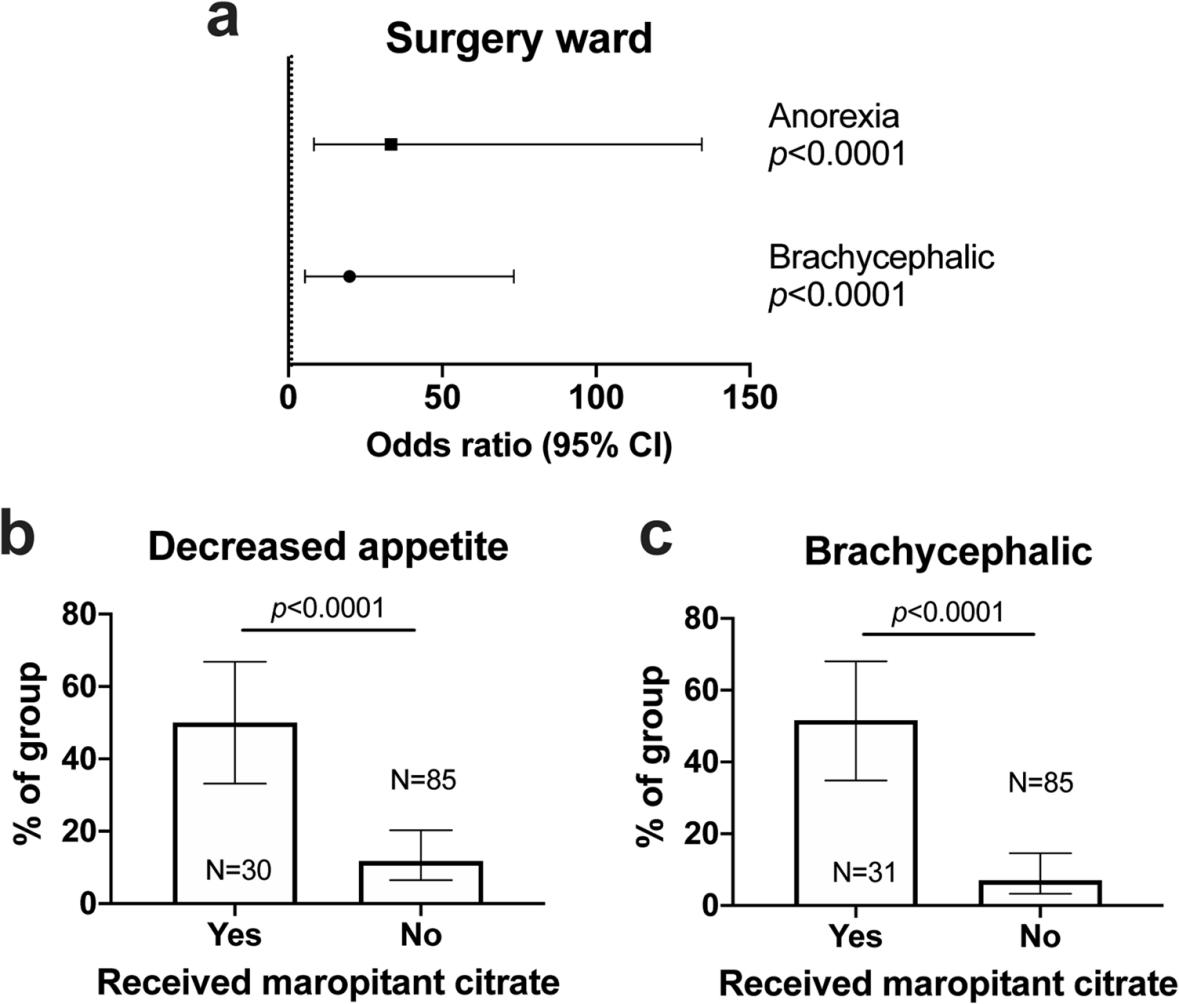
Membership of a brachycephalic breed and decreased appetite predict administration of maropitant in Surgery ward. (**a**) Forest plot showing odds ratios and 95% confidence intervals (CI) for variables significantly associated with maropitant administration in multivariable binary logistic regression analysis. Dashed line indicates threshold. (**b**) Proportion of dogs that had decreased appetite among those that did or did not receive maropitant. Bars show proportion with 95% confidence intervals; groups compared by Chi squared test. (**c**) Proportion of dogs belonging to a brachycephalic breed among those that did or did not receive maropitant. Bars show proportion with 95% confidence intervals; groups compared by Chi squared test.

As with omeprazole, we found maropitant was often administered to dogs presented with signs of vomiting, regurgitation, or nausea in both wards (Medicine 78/239, 32.6%; Surgery 28/144, 19.4%, Chi squared 7.81, p = 0.005). Examining those cases receiving maropitant without a history of such signs, the most common indications stated by clinicians in questionnaires are shown in Table 6. Among the concurrent diseases causing administration in Medicine ward were pancreatitis (n = 3) and azotaemia (1), compared to BOAS (n = 9) or vestibular syndrome/nystagmus (2) in Surgery. We again evaluated objective risk factors for administration of maropitant in both wards among this cohort of patients (Supplementary Tables S6–S8), finding that duration of hospitalisation and decreased appetite while hospitalised were significant predictors in a multivariable model for Medicine ward (Fig. 4a). As before, most dogs received maropitant early in their hospital stay (Fig. 4b), suggesting it acted as a marker of longer hospitalisation (Fig. 4c). The majority of dogs (20/33, 60.6%) observed to have a decreased appetite while hospitalised received maropitant at some point during their stay (Fig. 4d). In the Surgery ward (Fig. 5a), decreased appetite was also significantly associated with administration of maropitant (Fig. 5b), as was membership of a brachycephalic breed (Fig. 5c). These results demonstrated again that objective measures effectively recapitulated indications stated by clinicians for administration of maropitant, confirming this was chiefly related to decreased appetite/presence of nausea and, in Surgery ward, to concern about regurgitation and/or GOR in brachycephalic dogs.

**Table 6:** Stated indications for administration of maropitant in dogs with no history of GI signs on presentation.

Indication	Ward	
	Medicine (N = 21) N (%)	Surgery (N = 19) N (%)
Vomiting while hospitalised	3 (14.3)	3 (15.8)
Regurgitation while hospitalised	1 (4.8)	5 (26.3)
Diarrhoea while hospitalised	0	0
Decreased appetite while hospitalised	10 (47.6)	2 (10.5)
Signs of nausea while hospitalised	9 (42.9)	6 (31.6)
Signs of GI haemorrhage or ulceration	0	0
Concurrent disease	4 (19.0)	11 (57.9)
Concurrent medications	0	0
Prophylactic treatment to decrease risk of gastro-oesophageal reflux under GA	0	7 (36.8), all for dogs with BOAS

GA: general anaesthesia, BOAS: brachycephalic airway obstructive syndrome. Note that numbers summate to more than the number of questionnaire responses because some questionnaires contained multiple domains.

### Maropitant is almost always administered as recommended but often for indications incompatible with its known effects

Evaluating the drug and patient factors associated with administration of maropitant, we found it was almost always administered according to its marketing authorisation, with only 2 dogs receiving a dosage > 10% below and 1 dog > 10% above the recommended amount (Table 7). However, in the Surgery ward, a considerable proportion (15/28, 53.6%) of dogs received maropitant for the stated reason of treating or preventing regurgitation, which was not considered compatible with its known effects, and the same indication was stated occasionally in the Medicine ward (n = 2). Collectively, this showed that maropitant was almost always administered correctly but was often being used with the intention of treating or preventing regurgitation, for which purpose it is also not licensed in the UK.

**Table 7:** Frequency of drug and patient factors for inappropriate use of maropitant.

Factor	Ward	
	Medicine N with inappropriate use (%)	Surgery N with inappropriate use (%)
	N = 88	N = 53
*Drug factors*	1 (1.1)	3 (5.7)
Dose	1 (1.1)	3 (5.7)
Frequency	0	0
Route	0	0

	N = 49	N = 28
*Patient factors*	2 (4.1)	15 (53.6)
Regurgitation—treatment or prophylaxis	2 (4.1)	15 (53.6)

To investigate the attitudes underlying these decisions, we provided a case scenario to Surgery clinicians describing a dog with brachycephalic airway obstructive syndrome (BOAS) with intermittent regurgitation and ptyalism that is about to undergo surgery. In most cases (4/5), Surgery clinicians opted to administer both omeprazole and maropitant, with 1/5 choosing to administer omeprazole alone. Respondents could be split into those who indicated they believed this combination would decrease the risk of regurgitation during or after general anaesthesia (n = 3), and those who considered omeprazole would decrease the frequency or consequences of regurgitation while maropitant would alleviate nausea (n = 1), which was assumed to contribute to the GI signs. Taken together, this suggested there was a prevalent assumption among this group that maropitant would prevent regurgitation and/or nausea when published evidence does not support this notion.

## Discussion

Our study represents the first comprehensive investigation of the use of omeprazole and maropitant citrate in a veterinary hospital. We find that the most common drugs, omeprazole and maropitant, are administered to a large proportion of all admissions to our Medicine and Surgery wards, including to a considerable number of dogs that did not have GI signs before their presentation. The stated indications and risk factors for administration of each drug differ, but we find that development of GI signs while hospitalised is the major explanatory factor for use of omeprazole in such dogs. Assessing the use and stated indications for both drugs according to published materials, we find omeprazole is often administered outside its marketing authorisation, and that both drugs are frequently administered for purposes incompatible with their known effects in dogs.

The incidence and consequences of adverse effects associated with use of omeprazole and maropitant are unknown in veterinary medicine, but overprescribing of any drug increases the risk of prescribing errors and accidental overdoses, as well as imposing an unnecessary financial cost on the owners of the dog. Overprescribing of GP drugs is reported widely in human medicine, with known risk factors including incorrect diagnoses, overuse of PPIs for stress ulcer prophylaxis, and failure of deprescription in patients no longer needing treatment^12^. The occurrence of overprescribing and polypharmacy in veterinary medicine have rarely been investigated, but we suspect these practices are more likely to occur with drugs that are widely considered safe and affordable, as were omeprazole and maropitant among clinicians participating in this study. We believe this is likely to be a factor explaining the surprising rate of prescriptions, even among dogs not presented with any GI signs.

We identified that omeprazole was often administered to dogs with vomiting that had no complicating features, such as GI haemorrhage, ulceration, concurrent regurgitation, gastric mass lesions, or chronic signs that might indicate hypergastrinaemia. This was also the major reason why omeprazole was administered in hospitalised dogs that were not presented with GI signs. In some cases, this could have been intended as prophylaxis against the development of oesophagitis, but this complication appears to be rare when set against the high incidence of intermittent acute vomiting in dogs^28, 29^. Furthermore, it is not proven in such cases that administration of omeprazole would prevent oesophagitis. Consequently, guidelines in veterinary medicine recommend against routine administration of PPIs for cases with uncomplicated gastritis^4^, particularly because omeprazole causes considerable perturbation of the GI microbiome in dogs^30^ and people^31^. In cases where omeprazole is administered for suppression of gastric acid production, previous work indicates twice daily administration is probably necessary for a reliable and durable effect^32^. However, we found omeprazole was frequently being administered once daily, which was the recommendation published in older texts and formularies.

Maropitant was used most often for a stated indication of vomiting, which was considered appropriate for its known biological effects in dogs. However, it was also used frequently for nausea and decreased appetite, upon which signs it may have little effect depending on their origins. Although nausea is difficult to detect in dogs, previous studies have shown maropitant was not effective in decreasing nausea-associated behaviours after general anaesthesia^25^ or morphine premedication^22^, though it did decrease them less effectively than ondansetron after cisplatin treatment^26^. Of greater concern was the widespread use of maropitant for the stated indication of treating or preventing regurgitation. Maropitant is not licensed for this purpose in dogs, nor was it effective in preventing GOR in anaesthetised dogs when administered pre-operatively in a previous study^33^.

We did not intend to investigate the management of brachycephalic dogs with BOAS in this study, but dogs of such breeds were significantly more likely to receive omeprazole and maropitant in the Surgery ward for the stated reasons of decreasing the rate and/or consequences of regurgitation and GOR under anaesthesia. Both problems are common in brachycephalic dogs^34, 35^, though the prevalence of intra-operative GOR did not differ between brachycephalic and control dogs in a previous study^36^. Regurgitation in brachycephalic dogs is attributed principally to excessive negative pressure in the thorax^37^, but it is unclear whether affected dogs also experience nausea. Interestingly, owners reported ptyalism, which is often taken as a sign of nausea, to be much less common in such dogs than regurgitation^34^. Additionally, in our experience, brachycephalic dogs with regurgitation rarely have a decreased appetite, which might be expected if they were experiencing nausea. Evidence against the notion of nausea as a contributory factor is also provided by the finding that treatment of BOAS by surgery in brachycephalic dogs was sufficient to alleviate the frequency of regurgitation in most, without any additional medical treatment^34^.

We believe widespread use of omeprazole and maropitant in brachycephalic dogs has been related to a previous study that concluded “medical treatment of upper GI disorders [using omeprazole and the prokinetic cisapride]… dramatically improves the prognosis following surgical treatment of brachycephalic obstructive airway disease”^38^: this conclusion has been repeated subsequently by others^39^. However, this study was a prospective case series, meaning that such conclusions could not be drawn in the absence of a control group. Furthermore, the authors found most dogs had sustained improvement of their GI signs after medical treatment was stopped, suggesting correction of the BOAS was the more important factor in resolving GI signs. Against this background, we suggest there is little indication for administering maropitant for peri-operative or long-term management of regurgitation or GOR in dogs with BOAS, but randomised controlled trials evaluating its effects on nausea and GOR in this population would be welcomed. In contrast to maropitant, there is evidence in non-brachycephalic dogs undergoing orthopaedic surgery that omeprazole decreases the frequency of oesophageal low pH events^5^, though a later study with esomeprazole did not confirm this effect^6^. Owing to this evidence, we considered prevention of GOR to be an appropriate indication for administration of omeprazole in our hospital, even though the cost: benefit balance is undetermined for this intervention.

Although data were collected prospectively in this study, resulting in a very low rate of missing objective data in the case registry and high confidence in its accuracy, the rate of completion of questionnaires was lower, which is a limitation for the study. Additionally, we would ideally have asked clinicians to complete a questionnaire at the moment when they decided to prescribe a GP or antiemetic drug. Instead, we allowed up to one week to send the questionnaire, which could have affected the accuracy of recall for clinicians in a busy clinic. Furthermore, since we assured clinicians of their anonymity, we do not know if some clinicians completed questionnaires more often than others, meaning the opinions of some clinicians could have exerted a stronger effect in the final results. However, we mitigated this risk by calibrating questionnaire responses against objective data where possible, finding excellent concordance between these modalities. Data were collected from the two wards at different times, but were analysed only after data collection was completed in both wards, meaning that no results were discussed before the Surgery ward was recruited to the study. The act of asking clinicians to state their indications for use of GPs and antiemetics could have altered their responses, producing an observer effect. While we considered this unavoidable to some extent, we took care to frame the study among clinicians as a non-judgemental exercise in clinical governance, of which the goal was to evaluate the hospital culture of prescribing rather than the actions of individuals. Owing to its setting in a single centre, it is not clear if the results of our study are applicable more widely in veterinary specialist care: further studies will be required to determine if patterns of prescribing are similar in other centres.

In conclusion, we find omeprazole and maropitant are used in a large proportion of all cases presented for veterinary specialist care by clinicians who considered them safe and who underestimated their financial cost. However, they were often used outside dosing recommendations and with stated aims that were unlikely to be achieved by the known biological effects of the drugs in dogs. The extent of this prescribing warrants interventions to reduce the rate of polypharmacy.

## Methods

### Study setting

All data collection was conducted in the (internal) Medicine and Surgery wards of a veterinary university teaching hospital, which only accepts referral cases from primary care veterinarians predominantly in the South-east of England. At any time, new cases are admitted by 2–4 residents in each ward, under the supervision of board-certified specialist veterinarians. Both wards are staffed by permanent veterinary nurses registered with the Royal College of Veterinary Surgeons, most of whom have additional specialist qualifications in their areas of responsibility.

### Study design

A prospective observational study was conducted, which consisted of the following sections described in further detail:

1. Prospective case registry for dogs hospitalised for > 24 h
2. Questionnaires administered contemporaneously to clinicians of dogs that received a GP or antiemetic drug during their hospitalisation
3. Synoptic cross-sectional questionnaire administered to all clinicians who had participated in Sect. 2

The study protocol was approved by the Royal Veterinary College clinical ethical review board (URN SR2017-1292). Human participants gave informed consent for participation in the study. All research was conducted in accordance with relevant guidelines and regulations at our institution. A copy of all questionnaires used in the study is shown in Supplementary Methods.

### Prospective case registry

All dogs admitted to Medicine ward between 6th August and 19th December 2018 (period of 19 weeks) and to Surgery ward between 15th July and 24th November 2019 (period of 19 weeks) were eligible for enrolment in the registry. The study was restricted to patients hospitalised for more than 24 h. For dogs hospitalised more than once in the same ward during the study period, data from the second and subsequent visits was not used in the analysis. A structured form was used to standardise primary data collection for each eligible dog. Four types of information were collected: unique hospitalisation number and signalment; clinical signs and procedures; drugs administered; and signs of stress or agitation. On the day of patient admission to the ward, each dog was assigned a form for data collection; these forms were maintained by the permanent nursing staff. At the time of a scheduled handover between nursing shifts in the afternoon, the forms were updated with clinical information for the preceding 24 h: this included occurrences of vomiting, haematemesis, melaena, haematochezia, diarrhoea, regurgitation, and anorexia/decreased appetite; procedures under general anaesthesia; administration of GP or antiemetic drugs, glucocorticoids, NSAIDs, and anticoagulants; and signs of stress, including barking or vocalisation, digging or jumping at front of kennel, aggressive behaviour, or withdrawal/nervousness. In this way, information was recorded for each hospitalised dog prospectively on a daily basis during its hospital stay by staff who were employed permanently in the same ward. The forms were checked by the project co-ordinator for completeness every day, and missing information was obtained directly or by sending e-mails. Upon patient discharge, the forms were filed until analysis was completed.

### Contemporaneous questionnaires

In every dog where a GP or antiemetic drug was administered in the hospital, the attending clinician was asked by the project co-ordinators by e-mail to complete a structured questionnaire within 1 week of the prescription. In this questionnaire, the clinician was asked to state the name, dosage, and dose frequency of the drugs being administered, the expected duration of the course, and their indications for administration. Importantly, clinicians were prompted to give their main indications for each drug separately and in their own words. The questionnaire could be completed online (via a link in the e-mail) or on paper. At the outset of the study, clinicians were assured of their anonymity for these questionnaires, meaning the study co-ordinators sent blanket reminder e-mails but were unable to follow up individual cases if not completed. Responses from these questionnaires were coded to relate similar terms together for description, e.g. “anorexia” and “inappetence” were placed in the same domain.

### Synoptic questionnaire

After all data collection was completed, all clinicians who participated in the preceding section were asked to complete a synoptic questionnaire containing questions about the general use of omeprazole and maropitant, including their perception of the safety of the drugs, their cost, and their possible adverse effects. In addition, case scenarios were provided to try to resolve areas of interest in the initial data analysis.

### Assessment of appropriate prescribing

For the drugs omeprazole and maropitant, a list of drug factors (dosage, frequency, route of administration) and patient factors (appropriate reasons for use) was compiled, with reference to the marketing authorisation for maropitant and to published guidelines for GPs (shown in Table 4)^4, 27^. Assessment of drug factors was undertaken by reviewing the hospital ward sheets for every dog. Assessment of patient factors was undertaken primarily from the questionnaire responses provided by attending clinicians. Where a response was ambiguous, e.g. “vomiting” as an indication for omeprazole, the clinical record was reviewed by one study co-ordinator to determine if additional factors would render the decision appropriate according to the stated criteria. This varied among cases but usually included review of the clinical history and results of imaging performed by board-certified specialists in diagnostic imaging.

### Statistical analysis

All analyses were conducted with proprietary software (SPSS version 24, IBM; GraphPad Prism version 8, GraphPad Software Inc). Variables were assessed for normality by visual inspection of histograms and Kolmogorov–Smirnov tests. Since all pertinent variables were non-normally distributed, central tendency is described by median with IQR. Associations between categorical variables were assessed using Chi squared tests, or Fisher exact tests if there were fewer than 5 cases in any cell. Differences in continuous variables between groups were compared using Mann–Whitney U tests. Kaplan–Meier curves were produced to show when omeprazole and maropitant were administered in the hospital stay.

For assessment of risk factors for administration of omeprazole or maropitant in Medicine or Surgery wards, candidate variables were first assessed in univariable binary logistic regression. Those variables associated with administration at p < 0.1 were considered in the multivariable model, which was constructed by stepwise backwards elimination of non-significant variables based on calculation of the likelihood ratio. Interactions among variables in final models were evaluated by introducing the interaction term; this was discarded if not significant. The performance of models was evaluated by inspecting the receiver operator characteristic (ROC) curve and calculating the area under it, and by considering the proportion of cases correctly classified by the model.

Depending on the question being asked, the number of dogs used in each part of the analysis differed and is explained below:

- Description of overall frequency of administration of GP and antiemetic drugs: all dogs.
- Reasons and risk factors for administration of omeprazole and maropitant in dogs not presented with GI signs: only those dogs that did not have vomiting, regurgitation, diarrhoea, melaena, or regurgitation among their presenting problems.
- Assessment of drug factors in prescriptions of omeprazole and maropitant: all dogs that received these drugs.
- Assessment of patient factors in prescriptions of omeprazole and maropitant: those dogs that received these drugs and where a clinician completed a questionnaire to indicate why they prescribed it.

## Supplementary information

Supplementary Information.
