# Supplementary material for: Prospective observational study of the use of omeprazole and maropitant citrate in veterinary specialist care
Source: Sci Rep. 2020 Sep 25;10:15727. doi: 10.1038/s41598-020-72950-3 (PMC7519060; doi:10.1038/s41598-020-72950-3)
Supplement: Supplementary file 1 — Supplementary Information. [file 41598_2020_72950_MOESM1_ESM.pdf]

Prospective observational study of the use of omeprazole and maropitant citrate in veterinary specialist care

Rachel McCormack, Louise Olley, Barbara Glanemann, and James W Swann

Supplementary Information

Supplementary Table S1: Reasons for presentation of dogs hospitalised for at least 24 hours in Medicine and Surgery wards during the study periods.

	Ward	
	Medicine (N, %)	Surgery (N, %)
Disease category	N=239	N=144
Immunological/haematological	39 (16.3)	2 (1.4)
Urological	39 (16.3)	6 (4.2)
Gastroenterological/Pancreatic	71 (29.7)	19 (13.2)
Hepatobiliary	18 (7.5)	9 (6.3)
Respiratory	22 (9.2)	23 (16.0)
Cardiac	5 (2.1)	6 (4.2)
Dermatological	2 (0.8)	17 (11.8)
Endocrine	24 (10.0)	1 (0.7)
Orthopaedic	3 (1.3)	53 (36.8)
Infectious	9 (3.8)	6 (4.2)
Neurological	4 (1.7)	1 (0.7)
Toxicological	2 (0.8)	0
Ophthalmic	1 (0.4)	1 (0.7)

Supplementary Table S2: Dosage and stated indications for use of ondansetron in dogs in Medicine and Surgery wards, in all dogs regardless of whether they had gastrointestinal signs before presentation.

		Ward	
		Medicine (N=141)	Surgery (N=144)
N (%)		41 (17.2)	1 (0.7)
		N=40	N=1
Dosage (mg/kg per day; median, IQR)		1.0 (1.0-1.0)	1.0
Frequency (N, %)			
	Once daily	0	0
	Twice daily	40 (100.0)	1 (100.0)
Route			
	Intravenous	39 (97.5)	1
	Per os	1 (2.5)	0
Indications (N, %)		N=19	Questionnaire not completed for this dog
	Decreased appetite	9 (47.4)	
	Nausea	8 (42.1)	
	Vomiting	5 (26.3)	
	Pancreatitis	1 (5.3)	
	Haemorrhagic gastroenteritis	1 (5.3)	
	Inflammatory bowel disease	1 (5.3)	
	Gastrointestinal ulceration	1 (5.3)	

For indications, note that numbers summate to more than the number of questionnaire responses because some questionnaires contained multiple domains. IQR: Inter-quartile range.

Supplementary Table S3: Results of univariable logistic regression for predicting administration of omeprazole in dogs in Medicine ward.

**Variable**	**Type**	**Odds ratio**	**95% confidence interval**	**P value**
Hospitalisation (days)	Continuous	1.70	1.40-2.07	<0.0001
Glucocorticoid administration	Categorical	2.17	0.97-4.86	0.060
NSAID administration	Categorical	0.40	0.09-1.85	0.242
Any procedure under GA	Categorical	0.74	0.35-1.55	0.425
Decreased appetite	Categorical	4.85	2.14-11.00	<0.0001
Signs of stress or agitation	Categorical	0.98	0.48-2.02	0.958
Vomiting or regurgitation while hospitalised	Categorical	11.42	2.92-44.72	<0.0001
Diarrhoea while hospitalised	Categorical	5.33	2.05-13.91	0.001
Brachycephalic breed	Categorical	2.74	0.79-9.52	0.113

NSAID: non-steroidal anti-inflammatory drug; GA: general anaesthesia

Supplementary Table S4: Results of univariable logistic regression for predicting administration of omeprazole in dogs in Surgery ward.

Variable	Type	Odds ratio	95% confidence interval	P value
Hospitalisation (days)	Continuous	0.96	0.80-1.14	0.633
Glucocorticoid administration	Categorical	4.12	0.36-46.78	0.255
NSAID administration	Categorical	0.46	0.21-1.01	0.054
Any procedure under GA	Categorical	2.08	0.23-19.29	0.518
Decreased appetite	Categorical	1.83	0.74-4.55	0.191
Signs of stress or agitation	Categorical	0.82	0.35-1.90	0.639
Vomiting or regurgitation while hospitalised	Categorical	6.29	1.83-21.68	0.004
Diarrhoea while hospitalised	Categorical	1.65	0.57-4.84	0.359
Brachycephalic breed	Categorical	11.13	3.68-33.61	<0.0001

NSAID: non-steroidal anti-inflammatory drug; GA: general anaesthesia

Supplementary Table S5: Model parameters for multivariable logistic regression models predicting administration of omeprazole in dogs in Medicine and Surgery wards

Model parameter	Ward	
	Medicine	Surgery
Number of dogs in analysis<br>Total (event/control)	160 (40/120)	116 (39/77)
Proportion of cases correctly classified<br>Total % (event/control)	80.0 (42.5/92.5)	80.2 (61.5/89.6)
Nagelkerke's R^2	0.420	0.327
Hosmer and Lemeshow test (p)	0.130	0.569
Area under receiver operator curve (95% confidence interval)	0.851 (0.780-0.922)	0.758 (0.656-0.860)

Supplementary Table S6: Results of univariable logistic regression for predicting administration of maropitant in dogs in Medicine ward.

Variable	Type	Odds ratio	95% confidence interval	P value
Hospitalisation (days)	Continuous	1.69	1.39-2.05	<0.0001
Glucocorticoid administration	Categorical	1.75	0.78-3.93	0.179
NSAID administration	Categorical	0.18	0.02-1.37	0.097
Any procedure under GA	Categorical	0.93	0.45-1.92	0.839
Decreased appetite	Categorical	7.84	3.38-18.17	<0.0001
Signs of stress or agitation	Categorical	0.93	0.45-1.90	0.835
Vomiting or regurgitation while hospitalised	Categorical	3.26	0.99-10.74	0.052
Diarrhoea while hospitalised	Categorical	3.20	1.24-8.22	0.016
Brachycephalic breed	Categorical	1.75	0.48-6.30	0.395

NSAID: non-steroidal anti-inflammatory drug; GA: general anaesthesia

Supplementary Table S7: Results of univariable logistic regression for predicting administration of maropitant in dogs in Surgery ward.

Variable	Type	Odds ratio	95% confidence interval	P value
Hospitalisation (days)	Continuous	1.00	0.83-1.21	0.984
Glucocorticoid administration	Categorical	5.79	0.51-66.29	0.158
NSAID administration	Categorical	0.27	0.11-0.64	0.003
Any procedure under GA	Categorical	[All cases did]		
Decreased appetite	Categorical	7.50	2.83-19.85	<0.0001
Signs of stress or agitation	Categorical	0.25	0.08-0.77	0.016
Vomiting or regurgitation while hospitalised	Categorical	1.11	0.32-3.84	0.868
Diarrhoea while hospitalised	Categorical	1.29	0.41-4.08	0.660
Brachycephalic breed	Categorical	14.04	4.73-41.71	<0.0001

NSAID: non-steroidal anti-inflammatory drug; GA: general anaesthesia

Supplementary Table S8: Model parameters for multivariable logistic regression models predicting administration of maropitant in dogs in Medicine and Surgery wards

Model parameter	Ward	
	Medicine	Surgery
Number of dogs in analysis Total (event/control)	160 (41/119)	115 (30/85)
Proportion of cases correctly classified Total % (event/control)	85.0 (51.2/96.6)	82.6 (86.7/81.2)
Nagelkerke's R^2	0.413	0.514
Hosmer and Lemeshow test (p)	0.868	0.944
Area under receiver operator curve (95% confidence interval)	0.830 (0.751-0.910)	0.860 (0.777-0.944)

Supplementary Methods

This document contains a copy of questionnaires used in the study.

1. **Questionnaire on drug indications, provided as a paper copy or hosted on institutional questionnaire site online.**

Signalment (or attach label)

Age (years): ________________
Breed: ________________
Sex: ________________
Your case number: ____________

Which gastroprotectant or antiemetic drug(s) has been prescribed for the patient?

Gastroprotectant or antiemetic drug	Dose (mg/kg)	Frequency	Expected duration of course (days)
1.			
2.			
3.			
4.			

Please indicate the main reason why each drug was prescribed:

1.

2.

3.

4.

2. Synoptic questionnaire on attitudes to use of omeprazole and maropitant. This questionnaire was hosted on an institutional questionnaire website; questions are numbered below with possible responses shown in square brackets.

1. In dogs where you have prescribed/administered omeprazole, what effect(s) were you trying to achieve?

   Please list all below.
   [Free text answer]

2. In dogs where you have prescribed/administered maropitant citrate, what effect(s) were you trying to achieve?

   Please list all below.
   [Free text answer]

3. How safe do you think the drug omeprazole is in dogs?

   [Scale of 1-7: 1=Very unsafe, 4=Neither safe nor unsafe, 7=Very safe]

4. How safe do you think the drug maropitant citrate is in dogs?

   [Scale of 1-7: 1=Very unsafe, 4=Neither safe nor unsafe, 7=Very safe]

5. For a 10 kg dog, how much does it cost to administer omeprazole intravenously for 1 day at a standard dosage of 1 mg/kg BID?

   [5 options: £5, £10, £15, £20, £25]

6. For a 10 kg dog, how much does it cost to administer maropitant citrate (Cerenia) intravenously for 1 day at a standard dosage of 1 mg/kg SID?

   [5 options: £5, £10, £15, £20, £25]

7. Are you aware of any potential side effects of the drug omeprazole in dogs?

   Please list any potential side effects below.
   [Free text answer]

8. Are you aware of any potential side effects of the drug maropitant citrate in dogs?

   Please list any potential side effects below.
   [Free text answer]

9. Are there any types of patient to which you would NOT administer omeprazole?

   [Free text answer]

10. Are there any types of patient to which you would NOT administer maropitant citrate?

    [Free text answer]

11. Is omeprazole licensed for use in dogs in the UK?
    [Yes/No]

12. Is maropitant citrate licensed for use in dogs in the UK?
    [Yes/No]

Did you complete previous questionnaire(s) for dogs hospitalised in:

a. Medicine ward → directs to case scenarios
b. Surgery ward → directs to case scenarios

Medicine

1. A 4 year-old female neutered Springer spaniel is presented to you for further investigation of stranguria and haematuria. You find the dog has multiple calculi (probably calcium oxalate) in the bladder and is kept in the hospital to undergo cystotomy. While hospitalised before and after surgery, the dog refuses all food.

   In this dog, would you administer:

   a. Omeprazole → please explain why
   b. Maropitant citrate (Cerenia) → please explain why
   c. Both→ please explain why
   d. Neither

2. A 10 year-old male neutered Shih tzu is presented to you with moderate abdominal pain and distension and a history of 4 days of near-complete anorexia. Abdominal ultrasound and PLI measurement confirm he has active pancreatitis with associated localised peritonitis.

   In this dog, would you administer:

   e. Omeprazole → please explain why
   f. Maropitant citrate (Cerenia) → please explain why
   g. Both→ please explain why
   h. Neither

Surgery

A 2 year-old male entire French bulldog is presented to you with a chronic and progressive history of stertorous breathing, with occasional ptyalism and regurgitation. On physical examination, you identify the dog has severe brachycephalic obstructive airway syndrome (BOAS) and the owner agrees with your recommendation for surgery. The dog is admitted to the surgery ward and will have his procedure tomorrow.

In this dog, would you administer:

a. Omeprazole → please explain why
b. Maropitant citrate (Cerenia) → please explain why
c. Both→ please explain why
d. Neither
